# Effect of *Magnetospirillum gryphiswaldense* on serum iron levels in mice

**Published:** 2012-09

**Authors:** T Setayesh, SF Mousavi, SD Siadat

**Affiliations:** 1 Microbiology Research Center & Department of Bacteriology, Pasteur Institute of Iran, Pasteur Ave., Tehran, Iran; 2 Microbiology Group, Islamic Azad University of Tonekabon, Tonekabon Iran

**Keywords:** *Magnetospirillum gryphiswaldense*, Serum Iron Level, Mice

## Abstract

**Background and Objectives:**

The Magnetotactic bacterium *Magnetospirillumgryphiswaldense* (MSR-1) mineralizes the magnetite (Fe_3_ O_4_) crystals and organizes a highly ordered intracellular structure, called the magnetosome. Iron transport system supports the biogenesis of magnetite. Although iron is an essential trace element for many metabolic pathways of the body, increase or decrease in iron will cause many diseases. Mice were infected by MSR-1 to study survival of bacteria in mice when injected by different routes. The aim of this study was to investigate whether bacterial magnetite formation could take up Fe^2+^ ions from the blood an animal model.

**Material and Methods:**

In this study, MSR-1 at a dose lower than LD_50_ in 200 µl volume of PBS buffer was injected as intravascular (i.v), peritoneal (i.p) and subcutaneous (s.c) in mice. Number of viable bacterial was determined in organs such as liver, spleen and lymph node by measuring colony-forming unit (CFU). Moreover, serum iron level was evaluated by using commercial kits.

**Results and Conclusion:**

According to CFU measurements, after 96 hours, mice can clear MSR-1 from their body with different routes of injection. We have also shown that MSR-1 bacteria can affect the blood iron level in mice. The serum iron level decreased from control level in the first 24 h after i.v injection (P< 0.05). Our research on optimizing the biological magnetic system is still continuing.

## INTRODUCTION

The magnetotactic α-proteobacterium, *Magnetospirillum gryphiswaldense* (MSR-1) is a gram-negative, motile, aquatic and heterotrophic (1). Bacteria require a large quantity of iron to synthesize intracellular magnetic particles that termed magnetosomes, biomineralizes up to 100 cubo-octahedral magnetite (Fe_3_O_4_) crystals per cell, which is accompanied by the intracellular accumulation of tremendous amounts of iron (up to 4% of the dry weight) (2). This amount indicates that MSR-1 use very efficient systems to uptake, transport, and precipitation of iron that, however, are still poorly understood (3). On the basis of spectroscopic and biochemical analyses, it was suggested that for bacterial magnetite formation, Fe^3+^ was taken up from the environment and subsequently reduced intracellular 4^5^. A biochemical pool of iron is formed in the cells, essentially composed of ferritin and Fe^2+^ (6). Magnetite biomineralization proceeds first by transport of Fe^2+^ ions and ferritin into invaginated magnetosome vesicles where Fe^2+^ and Fe^3+^ ions co-precipitate (7). Final magnetite growth then occurs in fully formed mature magnetosomes (6, 7).

Although iron is the mineral element that is essential for microbial growth and is also essential trace element for many metabolic pathways in body, increase or decrease of iron will cause many disorders. These complications fall into two main groups. In primary haemochromatosis the iron overload is a consequence of a breakdown of a ‹‹switch›› in the gut which controls the uptake of iron. In secondary haemochromatosis the excess iron results from multiple blood transfusions administered because of a genetic blood disease (8, 9).

As a matter of fact, the unique crystalline and magnetic property of magnetosomes in Magnetospirillum gryphiswaldense has brought this entity into the focus of multidisciplinary interest as they are used in biomedical applications (10). The aim of this study was to survey of effect of bacterial magnetite formation that takes up Fe^2+^ ions from the blood in animal model.

## MATERIALS AND METHODS

### Bacteria strain and culture condition

*Magnetospirillum gryphiswaldense MSR-1* (DSM 6361) was purchased from Deutsche Sammlung von Mikro organism und Zellkulturen. The bacteria were grown at 28°C with modified *Magnetospirillum* growth medium (11, 12) that containing 500 µM ferric citrate as before described (12).

### Preparing mice

Female BALB/c mice obtained from the Animal Department of Pasteur Institute of Iran, Tehran, Iran, weighing 18-24 g divided into 3 different groups for injection as intravascular (i.v), peritoneal (i.p) and subcutaneous (s.c). Eeach group had at least 4 different times of incubation (24h, 48h, 72h & 96h) (n = 36) and control groups (n = 9). Mice were placed in polypropylene cages with stainless steel lids at an ambient temperature of 25±2 °C with a 12 h light/dark period. The animals had free access to standard pellet chow and drinking water.

### Determination of Lethal dose (LD_50_)

The bacterial pellets were washed in PBS buffer, and additional dilutions were made in water to obtain different cell densities used to precisely calculate the LD_50_ dose. Then, 1 × 10^7^ to 1 × 10^12^ CFU of bacteria were injected in mice and monitored for survival for 10 days after infection.

### MSR-1 injection & Bacterial Clearance

MSR-1 (1 × 10^9^ CFU) were injected with 200 µl volume of PBS as intravascular, peritoneal and subcutaneous to mice; the animal were sacrificed in 24, 48, 72 & 96 hours after injection, and the spleen, liver and lymph nodes were aseptically removed from each animal separately. The samples were rinsed with 5 ml sterile PBS, weighed and homogenized, then centrifuged at 1,000 rpm for 5 min (13). To evaluate the bacteria burden, the tissues separately homogenized in 5 ml PBS. Serial dilutions of earth tissue extraet were spread on Magnetospirillum growth medium plates and the number of colonies was counted after incubated for 10 days at 37°C.

### Blood samples Collection

At indicated time point (24, 48 and 72 h) after i.v, i.p & s.c injection of MSR-1, the blood samples were collected by cardiac puncture into centrifuge tube (15). Collection of samples were done between 8-10am since serum iron levels is affected by the time of day among other parameters the serum iron level of each sample was determined by using commercial kit (Pars Azmoon, Tehran, Iran) that the iron kit sensitivity was 5µg/dl (16).

## RESULTS

### Bacteria growth

After *Magnetospirillum* growth medium (supplemented with ferric citrate) had been prepared, bacteria colonies were visible about 1 mm in 5-7 days on medium, the colonies had a white-to-creamy appearance.

### LD_50_ determination of MSR-1

Survival estimates of 10 mice per group until 10 days with five doses of MSR-1 as fallow: 1 × 10^6^, 10^7^, 10^8^, 10^9^, 10^10^, 10^11^to 10^12^ CFU per ml. The LD_50_ was determined as1 × 10^9^ CFU per ml. T-test was utilized for statistical analysis between groups. The LD_50_ determinations were repeated twice with 10 mice per dose of the bacteria.

### Clearance of bacteria

**Fig. 1:**
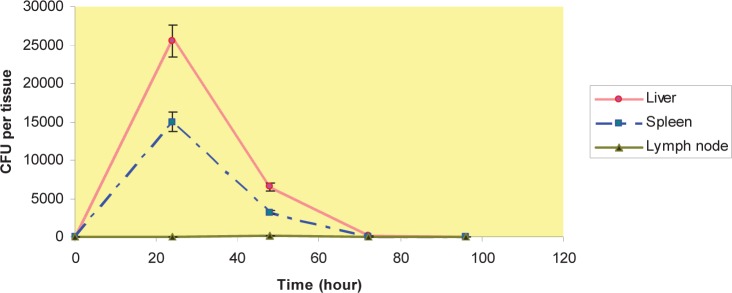
CFU per organ after i.v injection- 0, 24, 48, 72 & 96 hour after i.v injection, the number of colony forming units (CFU) in liver, spleen & lymph nodes.

**Fig. 2:**
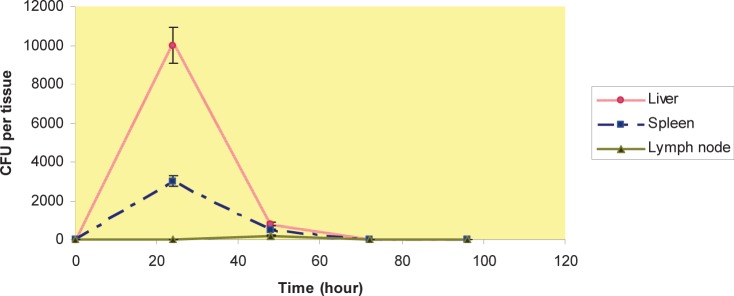
CFU per organ after i.p injection- 0, 24, 48 & 72 hour after i.p injection, the number of colony forming units (CFU) in liver, spleen & lymph nodes.

**Fig. 3:**
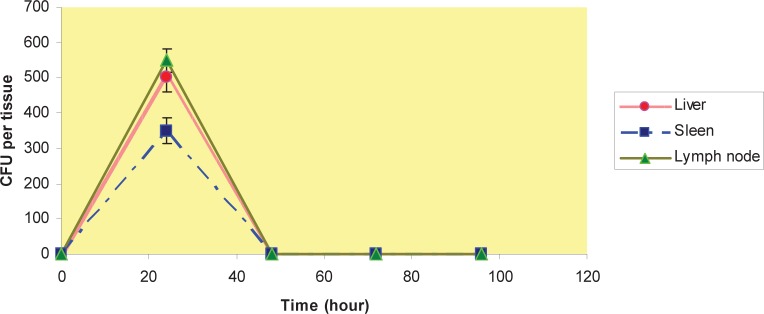
CFU per organ after s.c injection- 0, 24, 48 & 72 hour after s.c injection, the number of colony forming units(CFU) in liver, spleen & lymph nodes.

We had done a separate experiment with viable counts to investigate the distribution of MSR-1 for at last 96 hours. After 24 h period of i.v. injection, the numbers of CFUs recovered were higher in liver and spleen compared with lymph nodes. This trend reversed by hour 48, and 72, no viable bacteria were found in lymph nodes; The bacteria were found in liver and spleen in 48 and 72 h, and by hour 96 no viable bacteria were found in liver and spleen (Fig. 1); by hour 48 this trend changed and less viable bacteria were found in liver, spleen and lymph nodes after i.p and s.c injection compared with time point of 24h (Figs. 2 and 3). No viable bacteria were found in liver, spleen and lymph nodes after 72h (Figs. 1 and 2 & Fig. 3).

### Serum Iron level

**Fig. 4:**
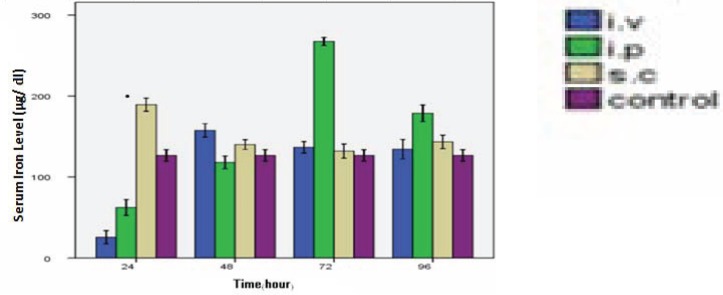
Serum iron level. Serum iron level was determined by using commercial kit after 24, 48, 72 & 96 hour; i.v, i.p & s.c injection.

The serum iron level has been decreased to 20% of control level in the first 24 h after i.v injection (P< 0.05). In contrast, after 48 h it has been deccreased over 100% of control level (P< 0.05) and after 2 h came back to normal level, there were not a significant different in serum iron level (P> 0.05). In i.p injection after the first 24 h there was a significant change in serum iron level (P< 0.05), in 48h it has been decreased a little compare with control level (P< 0.05) and after 72h it has been increased compare with control level (P< 0.05), and s.c injection there were significant changes (P< 0.05) (Fig. 4).

### Statistical analysis

In this study, t-tests were done for analysis serum iron level to compare differences between experimental and control mice. Statistical significance was determined by P < 0.05. The experiments results were repeated twice with a minimum of 3 mice per dose, group & day of the bacteria. However, the standard deviations (SD) was consistently <10% of the mean.

## DISCUSSION

Many previous studies have shown that, MSR-1 could uptake and transport iron from the environment (17). In this study we have reported effect of MSR-1 on serum iron level in mice. On the other hand, we propose to identify basic survey of phenomenon after MSR-1 injection.

The bacteria were injected into mice for LD_50_ determination (LD50 determination was needed for bacteria injection). After LD50 determination, bacteria were injected by i.v, i.p and s.c to identify the best effects of iron level changes and bacteria clearance in mice.

We have shown the role of bacteria on serum iron level by different routes of injection (i.v, i.p & s.c) in indicated time points (24, 48, 72, 96h) and also the CFUs measurements have shown that after 48 hours from i.p & s.c injection and 72 h after i.v injection, that mice could clear MSR-1 from body. As it is shown in our data, MSR-1 bacteria could be used to decrease iron levels in mice.

In summary, we suggest a new application for using magnetite characterization of MSR-1 in biomedicine. Our research on optimizing the biological magnetic system is still continuing.

Based on our data, we can suggest the survey of MSR-1 effect on iron overloaded diseases in animal models and also survey of mechanism of clearance and immunity system response to MSR-1.

In conclusion, we suggest that *Magnetospirillum gryphiswaldense* can uptake, transport, and precipitate iron in mouse and 72 hours after intake of *MSR-1*, it will be cleared.
